# Active Lumbar Spondylodiscitis on [^68^Ga]Ga-PSMA-11 PET/CT Mimicking Bone Metastasis

**DOI:** 10.3390/diagnostics13243616

**Published:** 2023-12-07

**Authors:** Florian Rosar, Caroline Burgard, Raffaele Gargiulo, Samer Ezziddin

**Affiliations:** Department of Nuclear Medicine, Saarland University, 66421 Homburg, Germany; caroline.burgard@uks.eu (C.B.); raffaele.gargiulo@uks.eu (R.G.); samer.ezziddin@uks.eu (S.E.)

**Keywords:** spondylodiscitis, PSMA, PET/CT, prostate cancer

## Abstract

We report a [^68^Ga]Ga-PSMA-11 positron emission tomography/computed tomography (PET/CT) scan of a 71-year-old man with metastatic castration-resistant prostate cancer (mCRPC) and concomitant active lumbar spondylodiscitis, both PSMA-positive on a PET/CT scan. This interesting image should advise colleagues to consider spondylodiscitis as a differential diagnosis of PSMA-positive findings in the spine, particularly if intervertebral space and soft tissue are involved.

**Figure 1:**
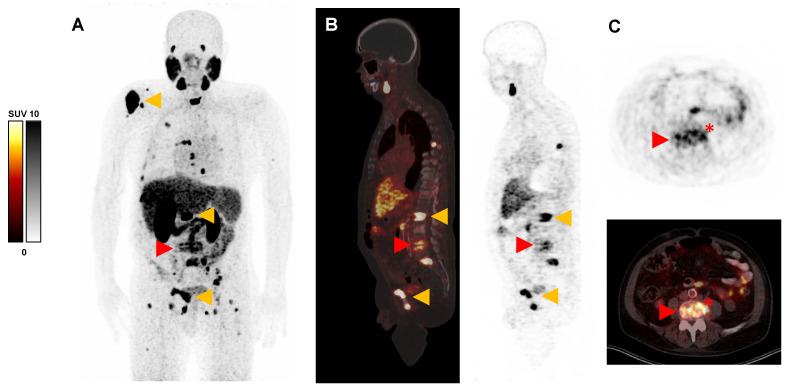
Prostate-specific membrane antigen (PSMA)-targeted positron emission tomography/computed tomography (PET/CT) scan with [^68^Ga]Ga-PSMA-11 (acquired 60 min post injection of 130 MBq) of a 71-year-old man with metastatic castration-resistant prostate cancer (mCRPC) referred for PSMA-targeted radioligand therapy. (**A**): Maximum intensity projection (MIP), (**B**): sagittal slices, and (**C**): transversal slices of [^68^Ga]Ga-PSMA-11 PET/CT, showing multiple intense PSMA-positive metastases (maximum standardized uptake value [SUVmax] up to 48.0, exemplary orange arrows) in the skeleton but also a moderate uptake (SUVmax 10.8) in the ground and upper plate of vertebral body L4 and L5, respectively (red arrow), also with involvement of the intervertebral space and surrounding soft tissue (red asterisk). Due to the distribution pattern, inflammatory etiology, i.e., spondylodiscitis, seemed more likely than additional metastasis, which could not be excluded at this point. Matching this differential diagnosis, the patient also reported newly developed pain in the lower back, and the inflammatory blood parameter, C-reactive protein (CRP), was highly elevated (61.8 mg/L). Neurological symptoms were not observed. Subsequently performed magnetic resonance imaging (MRI) confirmed the suspicion of active spondylodiscitis by showing low T1 and high T2 signal in disc space L4/5 and adjacent endplates, respectively. In contrast-enhanced T1 sequence vertebral endplates and paravertebral soft tissue revealed substantial enhancement. After the microbiological identification of streptococcus gallolyticus and subsequent appropriate antibiotic treatment, both pain and blood inflammation levels regressed. Spondylodiscitis mainly occurs in the elderly and is usually diagnosed on the basis of clinical, laboratory, and microbiological examinations, as well as imaging techniques such as CT, MRI, and PET with ^18^F-Fluorodeoxyglucose ([^18^F]FDG) [1,2,3,4,5]. In contrast, the finding of spondylodiscitis is rarely described on a PSMA PET/CT [6,7]. However, it is known that inflammatory processes may be related to the increased uptake of PSMA ligands, in line with our observed case of spondylodiscitis [8,9]. This interesting image should draw attention to spondylodiscitis as a differential diagnosis of PSMA-positive findings in the spine, especially when the intervertebral space and soft tissue are involved.

## Data Availability

The datasets used and analyzed in this paper are available from the corresponding author on reasonable request.
